# Family environment and frequency of vegetable consumption among children aged 6 to 12 years

**DOI:** 10.3389/fnut.2025.1552240

**Published:** 2025-10-08

**Authors:** Laura Raggio, Guadalupe Herrera, Adriana Gámbaro

**Affiliations:** ^1^School of Nutrition, Universidad de la República (UdelaR), Montevideo, Uruguay; ^2^School of Medicine, Universidad de la República (UdelaR), Montevideo, Uruguay; ^3^School of Humanities and Education Sciences, Universidad de la República (UdelaR), Montevideo, Uruguay; ^4^School of Chemistry, Universidad de la República (UdelaR), Montevideo, Uruguay

**Keywords:** parental influence, eating behavior, children, vegetables, frequency of consumption

## Abstract

**Introduction:**

Daily consumption of vegetables in recommended quantities is associated with health benefits. Its intake during the early years of life plays an essential role in the development of healthier eating habits. This study aims to contribute to the understanding of children’s eating behavior towards vegetables by exploring the frequency of consumption along with certain parental factors within the family environment of children between 6 and 12 years old.

**Methods:**

Factors such as nutritional knowledge (NK), frequency of vegetable consumption (FVC), interest in vegetable consumption, personal appreciation for vegetable consumption, and awareness of the health benefits associated with their consumption were examined.

**Results:**

The parental factors studied showed a positive correlation for some of the vegetables studied. Therefore, the necessity to study these factors individually rather than in groups is evident. Additionally, NK was found to influence the consumption of some of the vegetables studied.

**Discussion/Conclusion:**

Despite being aware of the health benefits associated with its intake, parents with lower levels of NK were shown to have lower FVCs and a greater willingness to increase their consumption. Therefore, increasing the parents’ FVC is necessary to improve their children’s FVC.

## Graphical abstract

**Figure:**
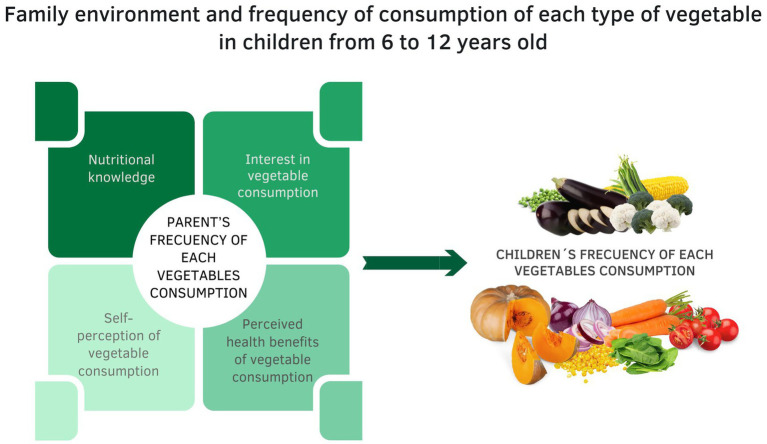

## 1 Introduction

Healthy nutrition is essential for human life, and it is built on a foundation of balance, variety, and moderation in the intake of required nutrients (1). To meet nutritional requirements, the World Health Organization (WHO) recommends a daily intake of 400 g of fruits and vegetables divided into five portions (2).

Vegetables not only contribute to preventing disorders caused by nutrient deficiencies, particularly micronutrients, but also reduce the risk of cardiovascular diseases (one of the leading causes of morbidity and mortality worldwide) and different types of cancers (3, 4). Thus, it is important to consume these foods daily in recommended quantities. Although promotional campaigns for fruit and vegetable consumption have been held in Uruguay in recent years, there remains a significant gap between dietary recommendations and the actual dietary intake. According to the most recent data, the average apparent consumption of fruits and vegetables in Uruguayan households is approximately 212 g per person per day, with quantities varying according to income levels (5).

This is relevant for the adult population, but it is still more alarming for children and adolescents. According to the same report, the apparent consumption of fruits and vegetables for children under 5 years old at the national level averages 127.7 g per person/day, with 59.8 g of fruits and 67.9 g of vegetables. In Montevideo, the consumption is 62.8 g of fruits and 72.0 g of vegetables, while in smaller towns and rural areas it is 56.1 g of fruits and 62.5 g of vegetables. The report also highlights that the consumption of fruits and vegetables varies according to the socio-educational level of the household. In middle-high socio-educational households, a greater variety of fruits and vegetables is consumed daily, whereas in middle-low socio-educational households, consumption is frequent but not daily and tends to focus on seasonal produce with less variety (5). Further emphasizing the concerning dietary habits, data from the recent Global School-Based Student Health Survey (GSHS), conducted in 2019 in Uruguay and recently published, evaluated the frequency of fruit and vegetable consumption among adolescents aged 13 to 17. Alarmingly, 8% of adolescents reported not consuming foods like lettuce, tomato, carrot, chard, or squash at all during the week prior to the survey. Furthermore, only 11.36% reported eating vegetables at least once a day during that period (6).

Vegetable consumption is relevant during childhood in the early years of life. Food preferences begin as early as 2–3 years of age (7, 8). Moreover, children who consume vegetables in recommended quantities have been associated with a healthier body weight during both childhood (9, 10) and adulthood (11). Due to vegetables typically having low energy density, increasing vegetable intake in children can improve their diet quality and decrease their energy intake (12, 13). Furthermore, the consumption of fiber-rich foods is associated with a reduced intake of calorie-dense foods (10).

Previous studies have assessed the existence of factors that influence food consumption, particularly fruit consumption, but few have focused on vegetables. Some of the factors include the consumer’s knowledge and beliefs, cost of foods, availability, and sensory characteristics (14–19).

In children, vegetable consumption is strongly influenced by parental factors, as they shape the dietary environment and behaviors within the household. These factors include parental knowledge, feeding practices, role modeling, and the accessibility of vegetables. Additionally, family-related factors such as daily exposure to one or several vegetables, homemade foods, and the overall obesogenic environment further impact children’s dietary habits (20–25). Understanding these aspects is essential for designing effective interventions to improve children’s vegetable intake.

The general interest in health expressed by consumers is a good predictor of dietary behavior (26, 27). Increasingly, consumers believe that foods contribute directly to their health (28). A way to assess consumers’ attitudes towards health is to utilize the General Health Interest (GHI) scale. GHI is a subscale of the “Questionnaire of attitudes upon health and taste” developed to assess consumers’ orientations toward the health and hedonic characteristics of foods (27). This approach has been used to assess general interest in health-related issues involving various types of foods, such as soy products (29), organic food (30), functional foods (31), and foods in general (32).

The influence of nutritional knowledge (NK) on dietary behavior has been extensively studied. Several studies have reported that higher NK is associated with increased intake of fruits and vegetables (33–38). However, other research has suggested that NK has little influence on food preferences and selection (39). Pearson et al. found that parents with a high intake of fruits and vegetables are more likely to have children with similar dietary habits (40). A recent review confirmed this positive correlation between parents and children (41).

The General Nutritional Knowledge Questionnaire (GNKQ), originally developed by Parmenter and Wardle (42), can be used to obtain the respondent’s NK level. Developed in the United Kingdom, the GNKQ has been adapted and validated in multiple countries to account for variations in dietary patterns and food availability. It was adapted for the Uruguayan population by modifying food-related items according to local consumption habits (36). The questionnaire typically covers various areas, including dietary patterns, nutrient sources, daily food choices, and the relationship between diet and disease. It has been validated and adapted for use in diverse populations to ensure cultural relevance and accuracy in assessing nutritional knowledge (43, 44).

Studies have consistently shown that demographic factors such as age, sex, educational level, and health status significantly influence NK scores. For instance, women and individuals with higher educational levels tend to score higher on the GNKQ (45–47). Additionally, research has shown that individuals with higher GNKQ scores tend to consume more fruits, vegetables, and low-fat products while reducing the intake of high-fat and high-sugar foods. A study conducted in Uruguay found that participants with greater nutritional knowledge reported higher consumption of healthy foods and lower intake of unhealthy options (36).

In children aged 6 and 12 years, growth needs are a priority, and it is noteworthy that during this period, food preferences become established and often persist into adulthood (48, 49). Therefore, it is crucial to examine the factors that influence these preferences in order to properly guide them towards a healthy, complete, and balanced nutrition throughout life (50).

This study aims to analyze the relationship between parental nutritional knowledge, attitudes, and the frequency of vegetable consumption among children aged 6–12 years in Uruguay.

## 2 Methodology

### 2.1 Participants

This cross-sectional observational study was conducted in Uruguay, involving several private educational centers in Montevideo city and surrounding areas. Initial contact was made with the principal of each center to inform them about the research. For those who agreed to participate, meetings were held to further explain the study and its methodology. An informative letter regarding the study was sent, followed by an invitation to join the database of parents with children between 6 and 12 years old. If parents had more than one child within this age range, they were asked to arbitrarily choose one child to complete the questionnaire about. A non-probability convenience sampling method was used. Parents were required to sign an informed consent form related to the study. The inclusion criteria included being a parent or legal guardian of a child aged 6 to 12 years enrolled in one of the participating education centers, having access to an electronic device to complete the questionnaire, and providing voluntary informed consent. The questionnaires were administered via electronic forms and sent to parents through email during the period from September 2015 to April 2016.

### 2.2 Questionnaires

Questionnaires were completed by the mother or father of the child between 6 and 12 years old. Parents responded to the following questionnaires in this order: (a) household characteristics; (b) NK level; (c) frequency of vegetable consumption (FVC) of the respondent parents and their children; and (d) interest in consuming vegetables, consumption appreciation, and the respondent’s health.

The parameters to be assessed through the questionnaire regarding household characteristics were the respondent’s gender (male/female); the respondent’s age; the highest level of education attained by the respondent subdivided into the following groups: level 1 (secondary school or lower), level 2 (incomplete university or technical education), and level 3 (university graduated); the respondent’s marital status, classified as “living with a partner” or “other life conditions”; the number of people in the home; the number of children under 12 years old in the home; and the number of adolescents aged 12 or older.

The General Nutritional Knowledge Questionnaire (GNKQ), used in this study consisted of 37 questions, comprising 106 items divided into four sections: (i) nutritional recommendations (11 items); (ii) content of nutrients of different foods (66 items); (iii) food selection (6 items); and (iv) health diseases or problems related to food (23 items). To perform the NK analysis, responses were dichotomized into a 0/1 variable, representing incorrect and correct answers, respectively. The highest NK total score that each respondent was able to accomplish was 106 (42).

Livingstone & Robson suggest that children aged eight and older can self-inform their intake of foods (51). Since this study assesses the frequency of consumption in children aged 6 to 12 years, it was decided not to use two different methodologies to evaluate the frequency of vegetable consumption by them. Therefore, the frequency will be reported by legal guardians for the entire study period. The frequency of consumption by both parents and their children was assessed in a single questionnaire.

Most research studies vegetables alongside fruits as a common group or refers to vegetables in general. It is noteworthy to remark that the present research studied the aforementioned factors for the 18 most commonly consumed vegetables in local markets. The questionnaire consisted of a list of said vegetables, which included: tomato, lettuce, carrot, beetroot, eggplant, zucchini, onion, cucumber, pumpkin, spinach, chard, pepper, cabbage, broccoli, green beans, corn, peas, and cauliflower. For each vegetable listed, the respondent was asked to indicate the frequency of personal consumption as well as their child’s consumption using a 7-point structured scale (1 = never, 2 = less than once a month, 3 = once or twice a month, 4 = several times a month, 5 = once or twice a week, 6 = several times a week, 7 = every day).

To assess parents’ attitudes towards health, an adapted part of the Health and Taste Attitudes Questionnaire, developed by Roininen (27), was used. The questionnaire consisted of statements on a 1–7 scale, where 1 = strong disagreement and 7 = strong agreement. Moreover, it was combined with the Food Choice Questionnaire (52), which includes items related to vegetable consumption.

### 2.3 Statistical analysis

The qualitative variables are presented in terms of their absolute frequencies and percentages. In the case of quantitative variables, summary measures are presented (median and standard deviation in the case of normal distribution).

The normality of the data distribution was assessed through the Shapiro–Wilk test and graphically through histograms.

For each respondent (father, mother, or legal guardian), the NK total score was calculated. Regarding the FVC of the 18 vegetables studied, the median and standard deviation were calculated. To compare the FVC between parents and children, a paired-sample student’s *t*-test was performed, and the consumption frequency association of parents and their children was estimated through Spearman’s rank correlation coefficient. Aiming to identify the underlying factors related to the questionnaire of interest in vegetable consumption, as well as consumption appreciation and the respondent’s health, an exploratory factor analysis (EFA) was applied. An EFA was performed using principal components extraction, without forcing, and with Oblimin rotation to estimate factor loadings in the constructs. Moreover, the internal consistency of each segment of interest for vegetable consumption, consumption appreciation, as well as the respondent’s health, was assessed through Cronbach’s alpha. The mean of the scores in each of the items that compose them was calculated, and it was determined to treat such segments as separate constructs. To characterize the studied population, the mean of each construct was calculated for all parents’ populations. Additionally, the association between parents’ NK level and the CVF of the 18 studied vegetables was assessed using Spearman’s rank correlation coefficient, along with its relationship to the three dimensions of the questionnaire: interest in vegetable consumption, appreciation of consumption, and health. Absolute values lower than 0.4 were considered weak correlations, values between 0.4 and 0.69 were classified as moderate, and values equal to or greater than 0.7 were classified as strong correlations.

To identify groups of parents, a Hierarchical Cluster Analysis (HCA) was conducted using Euclidean distance and Ward’s method on all questionnaire responses related to vegetable consumption appreciation, and health, with the classification forced into two clusters. The *t*-test was applied for independent sampling to evaluate the differences among the subsamples concerning the questionnaire of interest, consumption, and health, as well as for the FVC of each of the 18 vegetables studied, aiming to identify specific questionnaire items in which significant differences could be observed between groups. Additionally, household characteristics were evaluated using a chi-square test based on the groups generated.

According to the groups found, composed of parents with different interests in vegetable consumption, consumption appreciation, and health, the NK level was compared using the student’s *t*-test. Regarding the FVC of the 18 vegetables studied, the median and standard deviation were calculated. Furthermore, FVC among the groups of parents was calculated using the student’s *t*-test for independent sampling. A Spearman’s rank correlation coefficient was used to assess the association between the NK level of the parent group and the FVC of the 18 vegetables studied, as well as the three dimensions of the questionnaire. The significance threshold for the analyses was 0.05. Statistical analyses were performed through R version 4.0.4, XL-Stat 2021 software (Addinsoft, NY), and SPSS 19 testing version.

## 3 Results

### 3.1 Studied population (*n* = 162)

#### 3.1.1 Characterization of the respondent population

A total of 162 completed all questionnaires. The parents who formed the database were predominantly mothers (86%), aged between 30 and 45 years old (71%), with university education (80%), living with a partner (79%), and most homes had four people (51%).

The educational centers that participated in this study were private schools located in the capital city of Uruguay and its surrounding areas. This context suggests that the study population predominantly represents families from middle to upper-middle socio-economic strata.

There is a significant positive correlation, from moderate to high, between parents and children (Table 1). For the 18 vegetables studied, the responsible adults present higher consumption frequency than their children. The higher the frequency of vegetable consumption among the legal guardians, the higher it was among the children for the 18 vegetables studied. The most commonly consumed vegetables by both guardians and children were tomatoes, onions, lettuce, pepper, pumpkin, and carrot. In contrast, the least consumed were: cauliflower, eggplant, cucumber, broccoli, and green beans.

**Table 1:** Consumption frequency of parents and children for the 18 vegetables studied.

Vegetables	Parents		Children		Rs
	Mean	SD	Mean	SD	
Tomato	5.12	1.37	4.29	1.84	0.626**
Onion	4.78	1.43	4.11	1.70	0.709**
Lettuce	4.57	1.56	3.00	1.82	0.474**
Pepper	4.41	1.34	3.72	1.67	0.707**
Pumpkin	4.18	1.28	3.83	1.48	0.797**
Carrot	4.06	1.27	3.52	1.45	0.675**
Corn	3.94	1.22	3.79	1.38	0.769**
Zucchini	3.85	1.29	3.22	1.56	0.668**
Spinach	3.62	1.31	3.12	1.40	0.762**
Peas	3.58	1.17	3.19	1.38	0.718**
Chard	3.06	1.34	2.72	1.35	0.778**
Cabbage	2.72	1.39	1.98	1.22	0.550**
Beetroot	2.67	1.30	1.96	1.23	0.501**
Broccoli	2.60	1.36	2.20	1.30	0.724**
Eggplant	2.50	1.23	1.79	1.08	0.525**
Green bean	2.29	1.12	2.05	1.13	0.687**
Cucumber	2.25	1.32	1.80	1.26	0.566**
Cauliflower	1.62	0.97	1.40	0.80	0.724**

Md: median (between 1 and 7); SD: Standard Deviation; Rs: Spearman’s rank correlation coefficient. ** Significant at level 0.01.

#### 3.1.2 Exploratory factor analysis in the responses to the questionnaire of interest in vegetable consumption, consumption appreciation, and health of respondents

Through EFA of the 162 responses of the questionnaire, three dimensions were found that explained 58% of the variance, with communality values higher than 0.3 (Table 2). Dimension 1 was explained in items 7, 8, and 10 of the survey, which refer to the respondent’s interest in increasing their vegetable consumption (INTEREST). Dimension 2 was explained in items 3, 4, and 6 of the survey, which refer to the respondent’s appreciation of adequate consumption of vegetables (CONSUMPTION). Finally, dimension three was explained in items 1, 2, 5, and 9 of the questionnaires, which refer to vegetable consumption and its health benefits (HEALTH).

**Table 2:** Factorial loads in each axis, construct name, internacy consistency value, and the average of each construct for the total population surveyed.

Construct	1	2	3	Name	Cronbach’s alpha	Mean (*n* = 162)
1. Food is important for health.	−0.103	−0.164	**0.570**	Health	0.516	6.5
2. Vegetable consumption is important for health.	−0.091	0.124	**0.560**			
5. Vegetable consumption helps to prevent cancer.	0.113	0.138	**0.638**			
9. Vegetable consumption helps to prevent some cardiovascular diseases	0.319	0.006	**0.508**			
7. I’m interested in making foods with a greater amount of vegetables	**0.83**	0.172	−0.08	Interest	0.723	6.0
8. I’m interested in making foods with a greater amount of vegetables, although I do not like them much	**0.740**	−0.169	0.021			
10. I’m interested in improving my intake of vegetables.	**0.881**	−0.067	−0.011			
3. I consume a lot of vegetables	−0.042	**0.884**	−0.008	Consumption	0.775	5.3
4. My vegetable consumption is adequate.	−0.173	**0.919**	−0.062			
6. I care about the quantity of vegetables I intake with food.	0.315	**0.613**	0.244			

The internal validation assessment of the questionnaire revealed high internal consistency for the INTEREST and CONSUMPTION constructs, as evaluated according to the evaluation criteria described in the literature for Cronbach’s alpha (53). In the case of the HEALTH construct, the internal consistency found was moderate. From these results, it was determined that the segments of the form HEALTH, INTEREST, and CONSUMPTION should be treated as separate constructs. For each of these constructs, the mean of the items that constitute it was calculated, thus maintaining the original range of values of the questionnaire. These means for the respondent population can be observed in Table 3. The parents’ population reveals that they are aware of the existing relationship between vegetable consumption and its importance for health (6.5/7) and demonstrate a significant interest in increasing their vegetable consumption (6/7) while moderately expressing whether their vegetable consumption is adequate (5.3/7).

**Table 3:** A group of parents obtained from the surveyed population through the application of HCA upon the questionnaire’s results of: vegetable consumption, consumption appreciation, and respondents’ health.

Dimension	Questions	Cluster 1 (*n* = 86)		Cluster 2 (*n* = 76)		*p* value
		Median	SD	Median	SD	
Health	1. Food is important for my health	6.92	0.65	6.91	0.47	0.90
	2. Vegetable consumption es important for my health	6.85	0.69	6.91	0.44	0.51
	5. Vegetable consumption helps to prevent cancer	5.72	1.36	6.01	1.43	0.19
	9. Vegetable consumption helps to prevent some cardiovascular diseases	6.41	0.80	6.12	1.37	0.11
Interest	7. I’m interested in making foods with a higher amount of vegetables	6.37	0.98	6.37	1.14	0.98
	8. I’m interested in making foods with a higher amount of vegetables, although I do not like them much	5.91	1.41	5.13	1.76	0.003**
	10. I’m interested in increasing my vegetable consumption	6.41	0.97	5.99	1.45	0.03*
Consumption	3. I consume a lot of vegetables	4.34	1.10	6.21	0.88	<0.001**
	4. My vegetable consumption is adequate	3.71	1.15	5.89	1.01	<0.001**
	6. I care about the amount of vegetables that I intake with my foods	5.38	1.42	6.53	0.82	<0.001**

SD: Standard Deviation.

#### 3.1.3 Relation between parents and children’s NK and FVC and their interest in vegetable consumption, consumption appreciation, and health of the respondent

When analyzing the survey’s results regarding parents’ NK and FVC of the vegetables studied, a significant weak positive relationship was found for tomato (rs = 0.175), lettuce (rs = 0.158), and broccoli (rs = 0.196). Due to the significant and positive correlation between parents and children’s frequency of consumption, parents with a high level of NK will have higher consumption of these three vegetables and thus will their children.

For onion, pumpkin, chard, spinach, pepper, green beans, and peas (8 of the 18 vegetables studied), there was no relation found between the frequency of consumption of these vegetables and the three dimensions (HEALTH, INTEREST, and CONSUMPTION). On the other hand, for tomato (rs = 0.287), lettuce (rs = 0.273), carrot (rs = 0.162), beetroot (rs = 0.264), eggplant (rs = 0.261), zucchini (rs = 0.164), cucumber (rs = 0.175), cabbage (rs = 0.301), corn (rs = 0.195), and cauliflower (rs = 0.258), a slight and significant positive correlation was found between the frequency of consumption of each of the vegetables aforementioned and the CONSUMPTION dimension. The greater the appreciation for vegetable consumption as adequate, the higher the frequency of vegetable intake, in which such correlation was observed. Broccoli was the only vegetable, among those studied, that presented a significant positive correlation, albeit small, in the health dimension (rs = 0.204). Therefore, the greatest appreciation of positive consumption of broccoli is associated with a higher frequency of consumption.

### 3.2 Group of parents in the surveyed population

The Hierarchical Conglomerate Analysis (HCA) allowed us to identify two significantly distinct clusters of parents (Table 3). Said groups were characterized as follows:

Cluster 1 (*n* = 86) shows an interest in increasing their consumption. However, they do not like vegetables much (item 8), and they do not consume large quantities of vegetables (item 3). Furthermore, they are also aware that they do not have an adequate consumption (item 4). Still, they are interested in the quantity of vegetable consumption (item 6).

Cluster 2 (*n* = 76) shows interest in the quantity of vegetables they consume (item 6) and considers their consumption adequate (item 4); therefore, they present a lower interest in increasing their vegetable consumption (item 8), which is consistent with the previous responses.

Both groups considered that vegetable consumption is important for health and disease prevention, with no significant differences for any of the items 1, 2, 5, or 9 (items that describe the HEALTH dimension). Moreover, both clusters are highly interested in preparing foods with greater amounts of vegetables and increasing their vegetable consumption, with no significant differences for items 7 and 10 (some of the items that describe the INTEREST factor).

On the contrary, significant differences (*p* < 0.001) were found between items related to the CONSUMPTION dimension. In this case, the subjective factors are related to the respondent’s consumption and their perception of the consumption. Moreover, some differences were found (*p* < 0.05) in items 8 and 10, which comprise the INTEREST dimension.

#### 3.2.1 Characterization of the family groups found

Among the identified groups of parents, no statistical differences were found in the household characteristics assessed using the chi-square test.

Regarding the NK level of each group of parents, Cluster 2 (*n* = 76) had a mean of 74.2/106, while Cluster 1 (*n* = 86) had a mean of 70.8/106. Significant differences were found (*p* < 0.05) between these means.

As depicted in Table 4, the median and interquartile range FVC values were observed for the 18 vegetables in groups of parents. Significant differences were found between the Cluster 1 and Cluster 2 groups in the consumption frequency of tomatoes (*p* = 0.006), eggplant (*p* = 0.04), corn (*p* = 0.045), and cauliflower (*p* = 0.017) among parents. Regarding these four vegetables, the median consumption frequency among parents in Cluster 2 was higher than that of Cluster 1. For the children, significant differences were found in the consumption frequency of cucumber, cabbage, and cauliflower. For the three previously mentioned vegetables, the children’s frequency of consumption in Cluster 2 was higher than in Cluster 1.

**Table 4:** Consumption frequency of the 18 vegetables studied for parents and children, evaluating the differences between Cluster 1 and Cluster 2 subsamples.

Vegetables	Study group	Cluster 1 (*n* = 86)		Cluster 2 (*n* = 76)		*p*-value
		Median	SD	Median	SD	
Tomato	Parent	4.87	1.28	5.40	1.42	0.006**
	Child	4.01	4.00	4.61	1.74	0.050
r_**s**_		0.624**		0.579**		
Lettuce	Parent	4.40	1.50	4.76	1.61	0.128
	Child	2.85	1.82	3.17	1.82	0.231
r_s_		0.470**		0.465**		
Carrot	Parent	4.01	1.28	4.12	1.26	0.731
	Child	3.36	1.38	3.70	1.51	0.145
r_s_		0.666**		0.697**		
Beetroot	Parent	2.54	1.36	2.82	1.22	0.094
	Child	1.83	1.13	2.11	1.33	0.204
r_s_		0.463**		0.553**		
Eggplant	Parent	2.29	1.12	2.74	1.31	0.040*
	Child	1.59	0.89	2.01	1.23	0.013*
r_s_		0.500**		0.539**		
Zucchini	Parent	3.74	1.19	3.49	1.39	0.309
	Child	2.99	1.38	3.97	1.72	0.063
r_s_		0.461**		0.833**		
Onion	Parent	4.88	1.40	4.67	1.46	0.505
	Child	4.01	1.74	4.22	1.65	0.362
r_s_		0.663**		0.762**		
Cucumber	Parent	2.11	1.27	2.42	1.37	0.113
	Child	1.59	1.07	2.04	1.42	0.017*
r_s_		0.502**		0.609**		
Pumpkin	Parent	4.26	1.19	4.11	1.37	0.511
	Child	3.84	1.50	3.83	1.47	0.985
r_s_		0.776**		0.823**		
Spinach	Parent	3.74	1.35	3.50	1.27	0.193
	Child	3.17	1.44	3.05	1.36	0.663
r_s_		0.706**		0.829**		
Chard	Parent	3.19	1.32	2.91	1.35	0.172
	Child	2.76	1.38	2.67	1.33	0.731
r_s_		0.695**		0.879**		
Pepper	Parent	4.28	1.30	4.55	1.39	0,162
	Child	43.66	1.61	3.79	1.73	0.630
r_s_		0.668**		0.752**		
Cabbage	Parent	2.42	1.18	3.05	1.53	0.010*
	Child	1.76	1.03	2.22	1.36	0.020*
r_s_		0.575**		0.495**		
Broccoli	Parent	2.44	1.29	2.79	1.42	0.104
	Child	2.07	119	1.42	1.42	0.321
r_s_		0.747**		0.710**		
Green beans	Parent	2.51	1.16	2.47	1.08	0.876
	Child	2.00	1.13	2.11	1.14	0.514
r_s_		0.648**		0.737**		
Peas	Parent	3.57	1.16	3.59	1.19	0.838
	Child	3.11	1.45	33.28	1.30	0.300
r_s_		0.625**		0.835**		
Corn	Parent	3.79	1.16	4.12	1.26	0.045*
	Child	3.70	1.27	3.90	1.50	0.255
r_s_		0.763**		0.797**		
Cauliflower	Parent	1.49	0.90	1.78	1.03	0.017*
	Child	11.26	0.60	1.55	0.96	0.015*
r_s_		0.679**		0.731**		

SD: Standard Deviation; Rs: Spearman’s rank correlation coefficient. * Significant at level 0.05. Significant at level 0.01.

#### 3.2.2 Relation between NK, FVC of parents and children, and the interest in vegetable consumption, consumption appreciation, and health of respondents between Clusters

Cluster 2 is integrated by people who scored higher in “my vegetable consumption is adequate” (item 4) and also present the highest level of NK. While Cluster 1 is integrated by people who do not consume a considerable amount of vegetables and present a lower level of NK, despite having an interest in increasing their consumption. Despite this, no significant correlations were found between the three dimensions of the questionnaire of interest in vegetable consumption, consumption appreciation, respondent health, and the level of NK.

When analyzing the relation between the FVC and the questionnaire of interest in vegetable consumption, consumption appreciation, and health of the respondent, only a slight positive and significant correlation was observed between broccoli consumption frequency (for father, mother, or legal guardian) and one of the items that form the HEALTH dimension for Cluster 2. Differently, in Cluster 1, none of the 18 vegetables studied was significantly correlated with the HEALTH compound variable.

## 4 Discussion

### 4.1 Characterization of studied population (*n* = 162)

The parents’ group showed a good NK level (mean 70 correct questions over 106). This result is similar to that obtained by Gámbaro et al. in a Uruguayan population of 270 individuals who achieved a 66% correct response rate (36). The NK mean level of the studied population was higher than that reported in other studies (33, 38, 42), which could be explained by the high level of education of the participants, as 80% were university-graduated professionals.

The consumption frequency results of parents and children are consistent with findings reported by various researchers. According to a recent revision of 37 studies, including meta-analysis on the influence of parents’ practices on their children’s eating habits, it was concluded that parents’ diets are very similar to those of children; therefore, there is a positive correlation between them (41). Applying a similar methodology for this investigation, in which parents reported on the portions of their children’s consumption, vegetable consumption between mothers and children was positively correlated (54). In addition, the results of the present investigation showed significant moderate to high positive correlations for the consumption frequency between responsible adults and children for the 18 studied vegetables. Simply by consuming vegetables in front of their children, parents contribute to the exposure of these foods in their children’s environment. Haß & Hartmann observed higher consumption in those children whose parents consume such foods in greater amounts (16). In the studied population, children in homes where parents consume vegetables frequently also have a high FVC. It was also clear that the parents surveyed consumed vegetables more frequently than their children did. Both Hart et al. (55) and Wardle et al. (22) agreed that maternal diet is often predictive of the family’s diet, and it has also been found that children’s vegetable intake is positively related to their mother’s FVC. This is reflected in the coincidence that the most frequently consumed vegetables by parents are also the most consumed by their children. Food preferences and consumption habits are developed in early years, but if not established at that stage, they are often absent in adulthood. Therefore, it is essential to promote healthy habits, such as vegetable consumption, during the early stages of life (56–58).

Despite the extensive literature supporting the fact that consuming vegetables in recommended quantities and quality generates health benefits (4, 59, 60), the WHO-recommended intake is not being met globally (61–63). This has encouraged the governments in recent years to prioritize the promotion of vegetable intake (64). The present study found that parents consider their vegetable consumption inadequate, despite having a great interest in increasing it and being aware of its importance. Prior research indicates that nutritional knowledge is a necessary factor but not sufficient to generate changes in consumers’ eating behaviors (65). This is consistent with the findings observed in the group of respondent parents, who demonstrated knowledge about the importance of vegetable consumption, but have not yet acquired the habit of consuming it. Given the evident relationship between parents’ and children’s vegetable consumption frequency regarding the vegetables studied, if parents have not acquired this habit, neither will their children.

### 4.2 Groups of parents in the surveyed population

Regarding the NK level, there is no conclusive evidence supporting a direct relationship between higher NK levels in parents and higher levels of FVC. Previous systematic reviews, such as that by Barbosa et al. (66), have shown mixed results, with only a minority of studies finding positive associations between NK and dietary intake, particularly regarding vegetable and fruit consumption. This aligns with the present findings, where the differentiation between parental groups was primarily based on their appreciation of their vegetable consumption and their interest in improving it, rather than direct measures of consumption aligned with recommendations. This suggests a potential bias in self-perception that may influence reported behaviors.

Nevertheless, parents with higher NK tend to exhibit more favorable consumption patterns, including a higher frequency of intake of various vegetables, as reflected in the differentiated behavior observed between parental groups. Importantly, these differences also appear to extend to their children, reinforcing the notion that parental dietary habits play a significant role in shaping children’s eating patterns. This supports the well-established idea that parents serve as key role models in the development of healthy dietary behaviors in children, both through their habits and through the food environments they create at home.

These findings reinforce the notion that while nutritional knowledge alone may not be sufficient to modify eating behavior, it remains a crucial facilitator when individuals are motivated to change (67). The combination of knowledge, self-awareness of inadequate consumption, and interest in improvement can be leveraged in interventions designed to enhance dietary patterns. Moreover, understanding the barriers and motivations that influence vegetable consumption among parents is essential, as these factors likely mediate the influence parents have on their children’s dietary intake. Recent research further underscores the significance of parental beliefs and skills in promoting healthy dietary habits in children (25).

Interventions focused on expanding parental knowledge about the benefits of vegetable consumption and on building strategies to translate this knowledge into daily practice are essential. The present study highlights the need for targeted actions to support parents in adopting healthier eating behaviors, which, in turn, could contribute to healthier dietary habits in their children.

In line with recent studies that have proposed conceptual frameworks to explore the influence of individual knowledge, attitudes, and behaviors on dietary patterns (68), the present findings suggest the possibility of developing a conceptual model focused on parental factors—such as nutritional knowledge, self-perceived consumption adequacy, and motivation to increase vegetable intake—within the family environment to better understand the determinants of vegetable consumption among adults.

In summary, promoting vegetable consumption in children requires empowering parents to act as positive role models and equipping them with tools and strategies to facilitate behavioral change. Future research should explore the underlying motivations and barriers that influence vegetable consumption in diverse populations and evaluate the effectiveness of interventions focused on modifying parental determinants of dietary behavior to indirectly improve children’s eating patterns.

## 5 Conclusion

This study contributes novel evidence in the Uruguayan context on the relationship between parental factors and the frequency of vegetable consumption (FVC) in children aged 6 to 12 years. By assessing individual vegetables rather than grouping them broadly, the results highlight important differences in parental perceptions, knowledge, and consumption patterns associated with specific vegetables.

Our findings indicate that parental factors—nutritional knowledge, interest in consumption, and perceived health benefits—showed positive associations with the FVC of certain vegetables, underscoring the importance of evaluating these factors on a vegetable-specific basis rather than through generalized categories, such as “fruits and vegetables.” Notably, nutritional knowledge was weakly but significantly associated with higher consumption of tomato, lettuce, and broccoli.

Moreover, parents with lower nutritional knowledge not only reported lower FVC but also expressed greater interest in increasing their vegetable intake, suggesting a potential motivational entry point for interventions. These findings reinforce the importance of considering both knowledge and attitudes in dietary interventions targeting families.

Finally, as parental behaviors play a key role in shaping children’s food environments, strategies to improve parental vegetable consumption could have a positive impact on children’s dietary habits. Future studies should expand on these findings, incorporating broader populations and examining how targeted interventions on specific vegetables can translate into improved dietary patterns in children.

### 5.1 Limitations

It is acknowledged that the use of a non-probabilistic sampling strategy and the recruitment of participants exclusively from private education centers represent a limitation of this study, which may affect the generalizability of the findings.

Parents may mirror their personal preferences in the responses for their children, thus making the reported vegetable consumption in children slightly biased (69). However, it is a methodology widely used due to the difficulty that school-age children and younger children normally have in responding to these kinds of questionnaires.

In the questionnaire, vegetable consumption, consumption appreciation, and health of the respondent parent are evaluated, which reflects the consumption appreciation of the respondent. Such consumption can be biased if a specific pathology is present and a specific diet is followed. The above-mentioned aspects were not included in the questionnaire; therefore, the results indicate a single consumption appreciation, which may not reflect adherence to recommended quantities.

The present investigation approached the relationship between family environment and vegetable consumption in children through a non-probabilistic sampling from private educational centers. This characteristic implies that the conclusions obtained are not generalizable to the overall population. The application of self-administered online questionnaires can also introduce bias, both through socially desirable responses (70, 71) and through the potential for individuals seeking information online to influence their responses.

Nonetheless, this study provides exploratory evidence on an under-researched group, contributing relevant insights despite its methodological limitations. The data provide a useful starting point for understanding parental influences on children’s eating behaviors, particularly in populations where direct access to children poses practical challenges. Moreover, the findings help generate new research questions that can be addressed in future studies with broader and more representative samples.

## Data Availability

The raw data supporting the conclusions of this article will be made available by the authors without undue reservation.
